# COVID-19 vaccination hesitancy in India: State of the nation and priorities for research

**DOI:** 10.1016/j.bbih.2021.100375

**Published:** 2021-10-19

**Authors:** Sneha Chandani, Deepti Jani, Pratap Kumar Sahu, Udichi Kataria, Shailendra Suryawanshi, Jagdish Khubchandani, Sanket Thorat, Sohan Chitlange, Dharmendra Sharma

**Affiliations:** a Dr. D. Y. Patil Institute of Pharmaceutical Sciences and Research, Pune, Maharashtra, 411018, India; b Babaria Institute of Pharmacy, Vadodara, Gujarat, 391240, India; c School of Pharmaceutical Sciences, Siksha O Anusandhan University, Bhubaneshwar, Orissa, 751003, India; d Geetanjali Institute of Pharmacy, Geetanjali University, Udaipur, Rajasthan, 313001, India; e KLE College of Pharmacy, Belagavi, KLE Academy of Higher Education and Research, Belagavi, Karnataka, 590010, India; f Department of Public Health Sciences, New Mexico State University, Las Cruces, NM, 88011, USA; g Dr. D. Y. Patil Homoeopathic Medical College and Research Center, Pune, Maharashtra, 411018, India

**Keywords:** COVID-19, Vaccine, Vaccination, Behavior, Perceptions, India

## Abstract

**Background:**

Few COVID-19 vaccines were anticipated in India in early 2021. However, little was known about COVID-19 vaccination acceptance among the public. We conducted a nationwide study to understand the public’s perception about COVID-19 vaccines in December 2020.

**Method:**

An online survey was deployed using a multi-item validated questionnaire via social media websites and networking platforms for adults in India. We asked participants about vaccination willingness, concerns about vaccination, and their sociodemographic characteristics.

**Results:**

Nationwide, 1638 participants from 27 states/union territories took the survey where the majority of the participant were males (55%), 18–30 years old (52%), urban dwellers (69%), college-educated (81%), without a history of COVID-19 infection (92%). More than a fifth were either unaware of the vaccines (20.63%) or were not sure if they will get the vaccine (27%), and 10% refused to obtain the vaccine. Almost 70% of the population had concerns regarding the vaccines. Statistically significant differences (p<0.01) in awareness about vaccine and acceptability were observed based on age, educational qualifications, and employment status.

**Conclusion:**

While the majority of Indians would accept the vaccine, given the large population of India, even a small proportion of hesitant individuals would translate to millions of unvaccinated individuals. Strategic measures and policy decisions to enhance the rate of COVID-19 vaccination should be continuously planned and implemented in India.

## 1 Introduction

India was one of the world’s worst-affected countries due to the COVID-19 pandemic. By August 2021, more than 30 million Indians were infected and almost half a million died of COVID-19 infections (Thiagarajan, 2021; World Health Organization, 2021). Similar to many other countries, COVID-19 vaccines were granted emergency use authorization in India in early 2021. However, vaccine rollout in India faced a complicated path with political polarization, vaccine shortage, misinformation and rumors, challenges with registration and appointments, just to name a few (Thiagarajan, 2021; Chakraborty et al., 2021). By July 2021, nearly 75 million individuals were fully vaccinated translating to only 5% of the Indian population being vaccinated (OurWorldinData, 2021). Amidst the raging wave of infections and the suboptimal pace of vaccinations, little was known about COVID-19 vaccination hesitancy in India. Regional assessments of people’s preference for the COVID-19 vaccine remain a key in designing strategies to counter vaccine refusal. However, there is a dearth of nationwide studies and reports in India on the public’s perceptions of COVID-19 vaccination preferences, willingness, and concerns. Thus, the purpose of this analysis is to share results from one of the earliest and largest nationwide assessments on COVID-19 vaccination willingness and perspectives of the general public in India.

## 2 Materials and methods

An expert panel of scholars across multiple institutions was convened to design a valid and reliable questionnaire to assess COVID-19 vaccine-related knowledge, perceptions, and willingness in the adult Indian population. A comprehensive review of literature for existing studies on COVID-19 vaccination preference was conducted to create a draft questionnaire (Dror et al., 2020; Sallam, 2021). Subsequently, the team of experts convened for this study and other external experts reviewed the questionnaire to ensure face and content validity. The questionnaire was translated from English to Hindi, Marathi, Gujarati, and Odia to increase outreach to all regions of the country. Google forms were used to deploy the final questionnaire in December 2020. The questionnaire link was shared with a wide variety of community and social networks, and social media and networking platforms such as WhatsApp, Facebook, and LinkedIn. Adults (≥18 years of age) of Indian nationality were invited to participate. An a priori power analysis was conducted to estimate the required sample size for the study. Based on a 95% confidence level, a conservative 2.5% margin of error, and an estimated total adult population of 1 billion in India, 1537 adults were required to make adequate inferences to the total population of adults in India. The study protocols and procedures were approved by the Medical Ethics Committee at the D. Y. Patil Homoeopathic Medical College and Research Centre, Pune, Maharashtra, India (Approval: DYPHMCC/E−31/20)). Respondent consent was implied at the beginning of the study and potential respondents were informed that their participation in the study was voluntary and anonymous. Descriptive statistics (frequencies, percentages, and means) were computed to describe the study population and participants’ perspectives on COVID-19 vaccination. Inferential statistics (e.g, χ^2^ tests and regression analysis) were computed to assess group differences on COVID-19 vaccine preferences. Statistical significance for all tests and analyses was assumed a priori at p ​< ​0.05.

## 3 Result

**Fig. 1:**
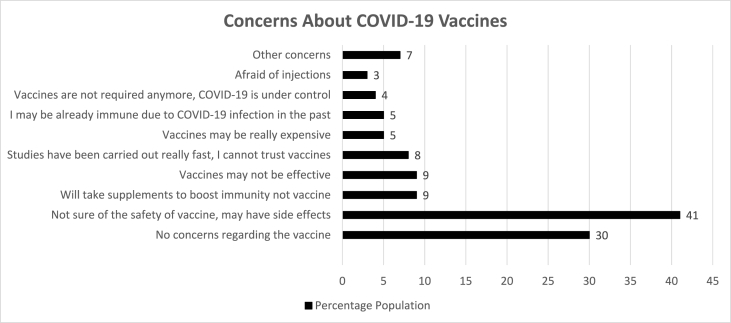
Concerns of study participants about COVID-19 vaccines.

A total of 1638 adults participated in the study where the majority were males (55%), 18–30 years old (52%), urban (69%), college-educated (81%), without a history of COVID-19 infection (92%) [Table 1]. Concerning COVID-19 vaccine hesitancy, when asked “if a vaccine is available for COVID-19, would you be willing to take it”, the majority of participants said ‘yes’ (63%) and more than a third (37%) were either ‘not sure’ (27%) or said ‘no’ (10%). The majority (71%) of the participants had at least one concern regarding COVID-19 vaccines. The most common concerns about the vaccines were related to safety and side effects, effectiveness, and rapid development of the vaccines [Fig. 1]. In group differences, compared to their counterparts, vaccine hesitancy (i.e., no or not sure) was statistically significantly higher among females (38%), urban dwellers (40%), full-time employed/salaried (41%), those with postgraduate education or higher (41%), those who had more than one concern about the vaccines (66%) or believed COVID-19 is not a severe disease (66%) and did not have COVID-19 infection in the past (37%). In a logistic regression analysis, compared to those who had no concerns about the vaccine, those who had one concern (OR ​= ​6.18, 95%Ci ​= ​5.40–9.63) or more than one concern (OR ​= ​10.80, 95%Ci ​= ​8.10–12.60) had statistically significantly higher odds of COVID-19 vaccination hesitancy. Despite adjusting for all the other sociodemographic characteristics (from Table 1), vaccination hesitancy odds remained statistically significantly higher among those who had one or more than one concern about the vaccines, compared to those who did not have any concerns.

**Table 1:** Characteristics of study participants and vaccination willingness.

Variable	N (%)	COVID-19 Vaccination Willingness			P value
		Yes	No	Not sure	
Total Population	1638(100)	1039(63)	158(10)	441(27)	
Age					0.005
18–24 years	758(46)	509(67)	53(7)	196(26)	
25–30 years	254 (16)	161(63)	28(11)	65(26)	
31–40 years	375 (23)	223(60)	46(12)	106(28)	
41–50 years	156(10)	83(53)	24(15)	49(31)	
≥51 years	95(6)	63(66)	7(7)	25(26)	
Gender					0.007
Male	906(55)	586(65)	100(11)	220(24)	
Female	732(45)	453(62)	58(8)	221(30)	
Residence Location					0.002
Urban	1130(69)	686(61)	121(11)	323(29)	
Rural	508(31)	353(70)	37(7)	118(23)	
Education					0.001
< ​College education	318(19)	226(71)	72(23)	20(6)	
College graduate	602(37)	388(65)	48(8)	166(28)	
Postgraduate and above	718(44)	425(59)	90(13)	203(28)	
Employment status					0.001
Student	669(41)	438(66)	51(8)	180(27)	
Self Employed/Business	108(7)	74(69)	16(15)	18(17)	
Employed/Salaried	695(42)	412(59)	84(12)	199(29)	
Unemployed	104(6)	67(64)	5(5)	32(31)	
Retired	32(2)	23(72)	0(0)	9(28)	
Other (e.g. daily wage earner)	30(2)	25(83)	2(7)	3(10)	
Perceived COVID-19 Severity					<0.001
Very severe	740(45)	510(69)	46(6)	184(25)	
Severe	713(44)	431(60)	76(11)	206(29)	
Somewhat severe	129(8)	75(58)	25(19)	29(23)	
Not severe	30(2)	10(33)	10(33)	10(33)	
No idea	26(2)	13(50)	1(4)	12(46)	
Ever had COVID-19 infection					0.09
No	1508(92)	948(63)	149(10)	411(27)	
Yes	130(8)	91(70)	9(7)	30(23)	
Vaccine related concerns (score)					<0.001
No concerns	481(29)	462(96)	2 (0.4)	17(4)	
One concern	953(58)	507(53)	104(11)	342(36)	
More than one concern	204(13)	70(34)	52(26)	82(40)	

N ​= ​1638, % may not add to 100 due to rounding off. p value indicates statistical significance for group differences assessed by Chi-Square tests.

## 4 Discussion

Data in this study indicate that more than a third (37%) of participants were either not sure about or refused to obtain the COVID-19 vaccines translating to roughly more than 200 million adults across the country. This rate of COVID-19 vaccine hesitancy is highly concerning given the threat of emerging variants around the world and a healthcare system in India that can be quickly overwhelmed with future outbreaks. Also, our estimates seem to be very conservative given that the sample was predominantly comprised of college-educated and urban individuals. A significant proportion of the Indian population is rural, without formal education, and affected by a greater digital divide (Thiagarajan, 2021; Chakraborty et al., 2021; Shah, 2021). This would mean that the actual rate of vaccine hesitancy could be much higher. A recent multisource report estimated that almost a third (29%–39%) of Indians were vaccine hesitant in early 2021 (Chowdhury et al., 2021). Another longitudinal study from January to June 2021 found that 12.7% of adult Indians would not obtain the COVID-19 vaccines (compared to the rate of 10% found in our study) (Umakanthan et al., 2021). Other reports have found that the major reasons for vaccine hesitancy and refusal in India are concerns about side effects and safety of the available vaccines (Chowdhury et al., 2021; Umakanthan et al., 2021; Danabal et al., 2021). These reports provide credibility to our study results. However, a common limitation with all these estimates, as mentioned earlier, relates to sample characteristics (i.e., most reports do not have data on individuals who are rural dwellers with lower education or lack access to technology precluding participation in studies).

Most of the factors associated with vaccine hesitancy in the Indian population are well-identified in studies worldwide (Dror et al., 2020; Sallam, 2021; Khubchandani and Macias, 2021; SolísArce et al., 2021; Khubchandani et al., 2021a). For example, females and those who had greater concerns about the vaccine were more hesitant. In addition, those who did not perceive COVID-19 as a serious disease were more likely to be hesitant. Given the consistency of these findings from India with studies worldwide, aggressive public health measures should be implemented to reach out to these populations. Interestingly and paradoxically, urban, highly educated, and full-time employed individuals were more likely to say ‘no or not sure’ about the COVID-19 vaccine. Studies indicate that these populations were not affected heavily due to the disruption caused by the pandemic and those who were badly affected economically or have no income or employment, are more likely to accept the vaccine in the hope to get back to work and normal life (Dror et al., 2020; Khubchandani and Macias, 2021; SolísArce et al., 2021).

Based on the results of this study, a comprehensive review of the literature, and the fact that COVID-19 vaccination hesitancy is not a monolithic concept, the following six recommendations are offered as urgent priorities for research on COVID-19 vaccination hesitancy in India (Sallam, 2021; Shah, 2021; Chowdhury et al., 2021; Umakanthan et al., 2021; Danabal et al., 2021; Khubchandani and Macias, 2021; SolísArce et al., 2021; Khubchandani et al., 2021a, 2021b; Sahay and Mishra, 2021; Dey, 2021; Kumar et al., 2021; Tarfe, 2021). First, given the vast diversity in India, regional studies and polls should be undertaken using local languages and dialects to understand the COVID-19 vaccination preferences of individuals. Second, studies should be conducted at a variety of avenues (e.g., community gatherings and places of worship, private healthcare facilities, and public primary healthcare centres, etc.) using a wide variety of methods such as quick polls, community-based focus groups, and interviews, door-to-door assessments, qualitative and community-based participatory research, in addition to traditional survey methods. Third, local level surveillance, research, and data collection on COVID-19 seroprevalence along with vaccination rates among various demographic sections of the Indian population should be undertaken to understand disparities in vaccination rates and COVID-19 cases based on sociodemographic characteristics. Fourth, the concerns of the public, perceived barriers to vaccination, and issues of access should be comprehensively explored to design public health campaigns and strategies to improve vaccine uptake. Fifth, research on healthcare workers and vaccinators should be undertaken to understand vaccinators’ confidence, training to address concerns of the public, and to understand the concerns of the public when they decide to get vaccinated. Sixth, a large proportion of priority populations (e.g., healthcare workers) remain unvaccinated in India; COVID-19 vaccination-related mandates, incentives, policies, or protocols for such populations should be explored from a moral, ethical, and legal standpoint (Dey, 2021). Finally, awareness campaigns and communication about COVID-19 vaccination should be evidence-based, regionally focused, culturally tailored, multipronged, and should be undertaken by policymakers, healthcare professionals, regional trusted voices, and role models. Research on the most effective means to communicate about COVID-19 vaccination benefits, alleviating concerns, dispelling myths and misinformation, and rumor control should be undertaken, keeping in view the vastly diverse population across India (Sallam, 2021; Shah, 2021; Chowdhury et al., 2021; Umakanthan et al., 2021; Danabal et al., 2021; Khubchandani and Macias, 2021; SolísArce et al., 2021; Khubchandani et al., 2021a, 2021b; Sahay and Mishra, 2021; Dey, 2021; Kumar et al., 2021; Tarfe, 2021).

While ours is one of the first assessments on COVID-19 vaccination hesitancy in India, a very large and diverse nation like India needs greater exploration of this topic. A continuing trend across the world remains of an initial greater demand and lesser supply of COVID-19 vaccines, followed by greater supply and lesser demand, in part due to vaccination hesitancy. The COVID-19 vaccination rates may have gathered pace in India, still, there is a large variation in vaccination rates across regions. By October 2021, nearly half of the Indian population received at least one dose of COVID-19 vaccines (OurWorldinData, 2021). As the vaccination rates plateau, India will now grapple with COVID-19 resistant and hesitant groups. COVID-19 vaccination hesitancy should be viewed as a serious public health threat by the government and civil society. Even a modest rate of vaccine hesitancy in India could translate to millions of COVID-19 vaccination refusing people across the nation leading to emerging variants and regular outbreaks for a long time in the future.

## Funding

This research received no external funding.

## Declaration of competing interest

Authors have no conflicts of interest to declare.

The authors have no conflicts of interest to declare that are relevant to the content of this article.
